# Serum Insulin-Like Growth Factor-1 and Nitric Oxide Levels in Parkinson's Disease

**DOI:** 10.1155/2009/132464

**Published:** 2009-03-12

**Authors:** Deniz Tuncel, Fatma Inanc Tolun, Ismail Toru

**Affiliations:** ^1^Department of Neurology, Faculty of Medicine, Kahramanmaras Sutcu Imam University, 46050 Kahramanmaras, Turkey; ^2^Department of Biochemistry, Faculty of Medicine, Kahramanmaras Sutcu Imam University, 46050 Kahramanmaras, Turkey

## Abstract

The aim of this study was to investigate the role of circulating growth hormone (GH), insulin growth factor-1 (IGF-1), IGF binding protein-3 (IGFBP-3), and nitric oxide (NO) concentrations in the patients suffering from Parkinson's disease (PD). The study groups were consisted of 25 patients with PD and 25 matched healthy subjects as a control. The NO level of patients in PD group (2.3 ± 0.4 *μ*mol/L) was significantly lower than that in the control group (2.8 ± 0.6 *μ*mol/L) (*P:.011*). Although there were no statistically significant differences in the GH, IGF-1, and IGF BP-3 levels among the two groups, in this preliminary study, we found low NO and mildly elevated IGF-1 levels in the patients with PD. The results may be associated with adaptation or protective mechanisms in the neurodegenerative disease processes such as seen in the PD. Further studies should be carried out to confirm our results.

## 1. Introduction

Parkinson's disease (PD) is a common neurodegenerative disorder that can cause significant disability and decline in the quality of life. It is not a single entity simply resulting from a dopaminergic deficit, rather most likely caused by a combination of genetic predisposition and environmental factors. Mitochondrial dysfunction has been proposed as a general basic mechanism underlying the neurodegeneration seen in PD [1, 2]. This condition is related to increased free radical production, oxidative stress, and decreased ATP production, that leads to increased intracellular calcium concentration, excitotoxicity, and nitric oxide related cellular damage [1, 3, 4].

Nitric oxide (NO) has varied physiological roles in the nervous system, including morphogenesis and developmental synaptic plasticity. However, in the pathological conditions, it could contribute to the oxidative stress and neurodegeneration [4, 5]. Clinical findings, evidences from experimental models, and postmortem studies revealed a connection of NO with the selective degeneration of dopaminergic neurons in the PD [4, 6]. Formation of NO can be regulated via the GH-IGF-1 system, but the cellular mechanism for this regulation is unknown [7]. Growth hormone (GH) exerts much of its biological activity by stimulating the production of (IGF-1), which is synthesized in nearly every tissue including the central nervous system. Insulin and IGF-1 have metabolic, neurotrophic, neuromodulatory, and neuroendocrine important functions in the brain. Studies among older people suggest that the GH-IGF-1 axis activity is reduced with age [7–9]. Thus, IGF-1 deficiency may be involved in the pathogenesis of age-related neurodegenerative diseases and might be relevant to the etiology of PD [8–10].

To further reveal the pathophysiological processes that were seen in the PD, we aimed to investigate the role of circulating GH, IGF-1, IGFBP-3, and NO concentrations in the PD patients in comparison with healthy subjects.

## 2. Materials and Methods

After receiving approval from the Hospital's Ethics Committee, we recruited patients from the out-patient clinic of our Neurology Department. All subjects had signed written informed consent before participation. A neurologist performed physical examination of each patient and noted the patient history. All PD patients showed rest tremor, rigidity, bradykinesia, and postural instability. These features with any one had begun displayed asymmetry. Patients with Parkinsonism, stroke, and dementia were excluded from the study. The patients with PD and healthy control groups were consisted of nonsmokers without other neurodegenerative, chronic, or infectious diseases. There were 25 patients in the PD group and 25 matched healthy subjects in the control group. The revision of Hoehn and Yahr staging scale was used to estimate the disease severity [11]. All of the PD patients were under the treatment with anti-PD drugs.

Venous blood samples were collected at 8 AM and 9 AM after an overnight fasting period. Within 1 hour after blood taking, the blood was centrifuged at 4000 rpm for 10 minutes, and the serum was stored at −70°C until analyzed. Stored sera were assayed for IGF-1, IGFBP-3, and GH by an automated chemiluminescent assay system (IMMULITE 2000, Diagnostic Products Corp., Los Angeles, Calif, USA). Serum nitric oxide measurement was performed using the Griess method for detection of nitrite levels [12].

Statistical assessments were carried out with SPSS 10.0 packet program. All data were given as mean ± standard deviation (SD). The results were analyzed using the Mann-Whitney *U-*test. A *P-*value of less than .05 was considered statistically significant.

## 3. Results

There were 25 patients (20 males, 5 females) in the PD group with a mean age of 67.9 ± 9.4 years, and 25 healthy subjects (20 males, 5 females) in the control group with a mean age of 64.3 ± 8 years. Age and sex distributions of patients and control groups were similar (*P* > .05). The mean Hoehn and Yahr stage was 3, and the duration of PD was 4.9 ± 3.1 years (see Table 1). NO, IGF-1, and IGFBP-3 levels in serum of PD patients showed no correlation with the duration and severity of the disease (measured by the Hoehn and Yahr staging scale).

The NO level of patients in PD group (2.3 ± 0.4 *μ*mol/L) was significantly lower than that in the control group (2.8 ± 0.6 *μ*mol/L) (*P:.011*). However, there were no statistically significant differences in the GH, IGF-1, IGFBP-3 levels among the patient and the control group (*P* > .05) (see Table 2).

## 4. Discussion

Mitochondria are critical regulators of cell death and have been implicated as an important player in the death of dopaminergic neurons in PD. NO has been suggested to contribute, via impairment of mitochondrial function, to the neurodegenerative process [1, 3, 4]. It has previously been shown that formation of NO can be regulated via the GH-IGF-1 system, and this system may prevent the NO-mediated neuronal damage. But, the cellular mechanism for this regulation is unknown [9]. We found that the NO level of patients in the PD group was significantly lower than that in controls. Although statistically not significant, IGF-1 levels were mildly elevated in the patients with PD. These changes may be considered to reflect local adaptive and protective responses to an underlying pathological derangement [3, 5, 9, 13].

The excess of NO could contribute to the formation of free radicals that could be involved in the death of dopaminergic neurons, leading to the development of PD symptoms [4]. Studies on nitrite and nitrate measurements in the cerebrospinal fluid (CSF) of PD patients have been contradictory, as there was no change [14–16], increase [17, 18], or decrease [19, 20]. On the other hand, some studies showed that CSF and plasma nitrate levels did not correlate with age at onset, duration, scores of the unified Parkinson's disease rating scales, and Hoehn and Yahr staging in the patients group [15, 16]. NO-mediated neurotoxic and neuroprotective effects seem to be dependent on its oxidative/reductive status, and the exact contribution to neurodegeneration is still not completely understood. An involvement of NO in the protective effect may occur via adaptive mechanisms. Therefore, the excessive NO synthesized in the course of adaptation is stored against the detrimental effects [4–6, 13]. We found that the nitric oxide level of patients in the PD group was significantly reduced than that in controls. The possible explanations for these low levels may be a defective NO-dependent adaptation mechanism or the exhaustion of NO storage in the course of PD. However, we found no significant correlation between these levels and the duration of disease and Hoehn and Yahr staging.

Growth hormone exerts much of its biological activity by stimulating the production of IGF-1, which is synthesized in nearly every tissue including the central nervous system and bounds to IGFBP-3. The GH-IGF-1 axis activity is reduced with age. Various studies showed that declining levels of serum IGF-1 contribute to age-associated brain impairments and neurodegenerative diseases such as PD and Alzheimer's [9, 21]. Also, IGF-I has recently been found of potential therapeutically use in different neurodegenerative conditions. Substantia nigra is one of the regions in the human brain, where a considerable density of IGF-1 receptors is evident. IGF-1 increases the survival of neurons in the brain stem and rescues embryonic DA neurons from programmed cell death [9, 22, 23]. It is found that serum IGF-1 levels are variable in the different diseases [9]. There were no statistically significant differences in the IGF-1 and IGFBP-3 levels among our patient and control groups. After a pathological condition develops, IGF-1 levels increase to protect affected neurons and to adapt to an underlying pathological process. This mechanism may explain the mildly elevated IGF-1 levels [9].

Our study has some limitations; our patient group is rather small and had a male predominance. The results of this preliminary study must be confirmed in larger patient groups with different age and sex distributions.

As a conclusion, there are few data about the role of NO on IGF-1, IGFBP-3 gene expression, and serum levels in PD. In this preliminary study, we found that there were low NO and mildly elevated IGF-1 levels in the patients. The results may be associated with adaptation or protective mechanisms in the neurodegenerative diseases process such as PD. Further studies should be carried out to confirm these results.

## Figures and Tables

**Table 1:** Age, sex, and clinic features of the patients with PD and healthy controls.

	Patients	Controls
Age (years)		
mean ± SD	67.9 ± 9.4	64.3 ± 8
Sex		
* *Male (*n*)	20	20
* *Female (*n*)	5	5
Hoehn and Yahr score		
mean ± SD	3 ± 1	
Duration of illness		
men ± SD	4.9 ± 3.1	

**Table 2:** Nitric oxide, growth hormone, insulin-like growth factor-1, and insulin-like growth factor BP-3 levels of the patients with PD and healthy controls.

	Patients	Controls
Nitric oxide (*μ*mol/L)		
mean ± SD	2.3 ± 0, 4	2.8 ± 0.6*
Growth hormone (ng/mL)		
mean ± SD	0.9 ± 0.9	0.7 ± 1.2
Insulin-like growth factor-1 (ng/mL)		
(mean ± SD)	132 ± 42	113 ± 51
Insulin-like growth factor BP-3 (*μ*g/mL)		
(mean ± SD)	3.4 ± 0.8	3.7 ± 1

*P *:.011, P:.52, P:.087, P:.19, respectively.*
